# Improving Patient Outcomes Through Effective Hospital Administration: A Comprehensive Review

**DOI:** 10.7759/cureus.47731

**Published:** 2023-10-26

**Authors:** Deepak Bhati, Meena S Deogade, Deepika Kanyal

**Affiliations:** 1 Hospital Administration, School of Allied Health Sciences, Jawaharlal Nehru Medical College, Datta Meghe Institute of Higher Education & Research, Wardha, IND; 2 Ayurveda Pharmacology, All India Institute of Ayurveda, New Delhi, IND

**Keywords:** healthcare policies, emerging technologies, quality improvement, healthcare leadership, patient outcomes, hospital administration

## Abstract

This comprehensive review delves into the critical role of effective hospital administration in shaping patient outcomes within the healthcare ecosystem. Exploration of key components, strategies, measurement methodologies, and future trends elucidates the multifaceted nature of hospital administration. Key findings underscore the profound impact of administrative decisions and practices on patient safety, satisfaction, and overall well-being. The review highlights the importance of patient-centred care and interdisciplinary collaboration for enhancing patient outcomes. It emphasises the significance of data-driven measurement and benchmarking, which are instrumental in assessing hospital performance and fostering continuous improvement. Looking ahead, emerging technologies, evolving healthcare policies, and persistent challenges are drivers of change in healthcare administration. However, amidst these transformations, the overarching message remains consistent: effective hospital administration is integral to improving patient outcomes. The conclusion calls for a collective commitment from healthcare leaders and policymakers to prioritise the development of capable administrators, invest in technology, promote value-based care, and address healthcare disparities. This collaborative effort ensures that the pursuit of better patient outcomes remains at the forefront of healthcare administration, ultimately shaping the future of healthcare for generations to come.

## Introduction and background

Hospital administration is pivotal in the healthcare ecosystem, serving as the cornerstone of healthcare institutions worldwide. The management and organization of hospitals are critical components in ensuring the effective delivery of healthcare services. Hospital administrators oversee various aspects of hospital operations, from financial management and resource allocation to quality improvement and patient safety [1]. Over the years, the field of hospital administration has evolved significantly, adapting to the changing dynamics of the healthcare industry. Traditionally, it focused primarily on administrative and logistical functions. Still, recently, it has expanded its scope to encompass a broader spectrum of responsibilities, including patient-centered care, data-driven decision-making, and strategic planning [2].

The overarching goal of any healthcare system is to provide high-quality care that results in positive patient outcomes. Patient outcomes measure the effectiveness and success of healthcare interventions and services. These outcomes encompass a wide range of factors, including the patient's overall health, satisfaction with care, recovery, and, notably, the prevention of adverse events specific to their treatment or condition [3]. Improving patient outcomes is a moral imperative and a critical component of healthcare quality and performance evaluation. Hospitals and healthcare institutions are continually working to enhance patient outcomes, recognizing that they are intrinsically connected to the reputation and success of the institution, as well as the well-being of the community it serves [1].

This comprehensive review aims to delve into the multifaceted realm of hospital administration and its profound impact on patient outcomes. We aim to explore the intricate interplay between effective hospital administration practices and patient care quality. By examining various dimensions of hospital administration, we intend to provide a holistic view of how administrative decisions and strategies can influence patient health, safety, and overall satisfaction.

## Review

Key components of effective hospital administration

Leadership and Governance

**Figure 1:**
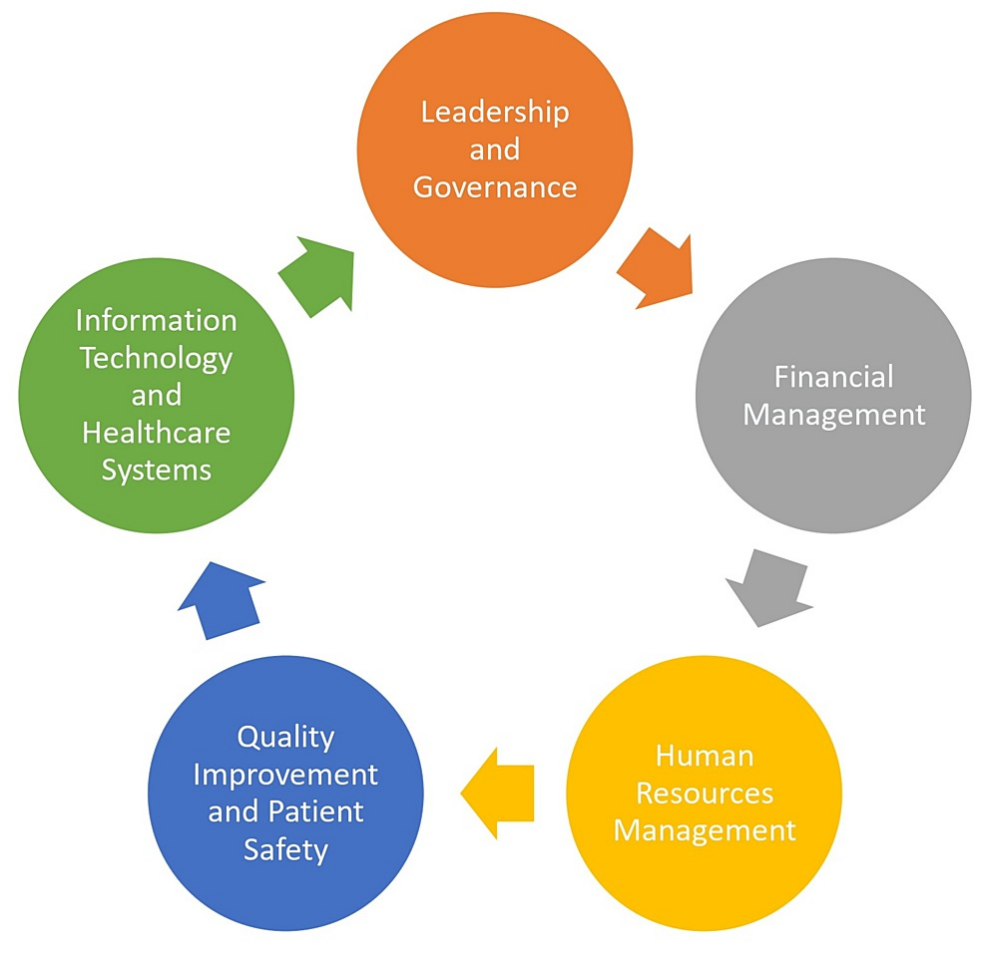
Key components of effective hospital administration

Effective hospital leadership and governance are foundational to achieving positive patient outcomes and ensuring the delivery of high-quality healthcare services. Hospital administrators, Chief executive officers (CEOs), and department heads play pivotal roles in shaping the organizational culture and establishing a framework for excellence in patient care [4]. Figure 1 shows the key components of effective hospital administration.

The role of hospital leadership in patient outcomes must be balanced. These leaders set the tone for the organization by exemplifying a commitment to patient-centered care. Their leadership style, vision, and values directly influence the behaviour and performance of healthcare staff. Strong leaders inspire and empower their teams, fostering a culture of collaboration, innovation, and accountability. They encourage healthcare providers to prioritize patient well-being, safety, and satisfaction in their work. Effective leadership also involves strategic planning, resource allocation, and decision-making that align with the hospital's mission to provide the best possible care [5].

The importance of governance structures in hospital administration is multifaceted and crucial in effective risk management. Governance encompasses the policies, procedures, and decision-making processes that guide the institution. In this context, effective risk management means identifying, assessing, and mitigating potential risks that could affect the hospital's operations, patients, and overall reputation. One way governance specifically affects this aspect is through clear lines of authority and responsibility. For example, a hospital with a well-defined governance structure ensures that designated individuals or committees are responsible for monitoring and addressing various risks. These could include clinical risks related to patient safety, financial risks, or compliance risks with regulatory standards. With established governance, these risks can be identified and managed more effectively [6].

To elaborate further, consider a scenario where a hospital's governance structure designates a risk management committee. This committee is responsible for assessing potential risks, such as medication errors, and developing strategies to minimize them. This way, governance ensures that risk management efforts are organized and systematic. Moreover, governance structures also promote adaptability in healthcare institutions. For instance, a robust governance framework in an ever-evolving healthcare landscape with changing regulations and emerging patient needs allows hospitals to quickly adapt and implement necessary changes in their risk management strategies. It ensures that the hospital can respond efficiently to new challenges and opportunities while maintaining patient safety and compliance with regulatory standards [6].

Ethical considerations are fundamental in the realm of hospital administration, influencing decision-making and practices that have a direct bearing on various aspects mentioned earlier. Ethical dilemmas can manifest in many healthcare domains, including patient confidentiality, the process of informed consent, end-of-life care decisions, and the equitable allocation of limited resources. Hospital administrators find themselves navigating these intricate challenges while steadfastly upholding the principles of beneficence (the promotion of well-being), non-maleficence (the prevention of harm), respect for patient autonomy (honouring patient choices), and the pursuit of justice in resource allocation. Ethical healthcare leadership necessitates a profound understanding of cultural diversity, a deep-seated respect for patient rights, and a dedicated commitment to ethical decision-making frameworks that unequivocally prioritize the welfare and well-being of patients [7].

Financial Management

Financial management plays a pivotal role in hospital administration, serving as the cornerstone for delivering quality care and the long-term sustainability of healthcare services. Within this dynamic landscape, hospital administrators are tasked with efficiently allocating resources, upholding cost-effectiveness, and maximizing revenue generation, all of which directly and profoundly influence patient outcomes [8]. To achieve these objectives, administrators must skillfully balance budgets, prioritize investments in healthcare technologies and personnel, and adopt innovative financial strategies. They need to ensure that each financial decision positively impacts patient care by improving the quality of services provided and maintaining accessibility and affordability for patients. Moreover, effective financial management is essential for fostering partnerships, driving research and development, and ultimately bolstering the overall performance of healthcare institutions [8].

Budgeting and resource allocation are foundational to effective financial management in hospitals. Administrators play a pivotal role in developing and managing budgets that align with the hospital's strategic goals. Strategic budgeting entails allocating resources to areas with the most significant impact on patient outcomes. This includes decisions related to staffing levels, acquiring and maintaining essential equipment, and investing in patient safety initiatives. By prioritizing resource allocation based on the needs of patients, hospitals can ensure that they are equipped to deliver high-quality care while making the most of their financial resources [9].

Cost-effectiveness and financial sustainability are pivotal considerations in healthcare financial management. Hospitals face the ongoing challenge of striking a delicate balance between controlling costs and upholding the quality of patient care. Healthcare administrators are entrusted with implementing cost-containment strategies that safeguard patient safety and maintain high care standards. To elaborate on this idea, achieving these goals may involve several key strategies, focusing on optimizing supply chain management, negotiating favourable vendor contracts, and introducing process improvements to minimize wastage. Hospitals can adopt efficient inventory control systems to optimize supply chain management, enhance demand forecasting, and streamline procurement processes. Hospitals can reduce unnecessary storage costs and minimize the risk of shortages that could compromise patient care by ensuring that essential medical supplies and equipment are readily available when needed. Effective supply chain management can also involve negotiating favourable vendor agreements to secure competitive pricing and quality assurances, ultimately driving down costs [10].

In parallel, process improvements can target waste reduction, such as minimizing redundant administrative tasks and ensuring that clinical workflows are as efficient as possible. These improvements save resources and enhance the overall patient experience by reducing delays and increasing the time healthcare professionals can devote to direct patient care. Achieving financial sustainability is a long-term commitment that ensures the hospital can continue serving the community and invest in improving patient outcomes. A financially stable hospital is well-positioned to make strategic investments in infrastructure, technology, and workforce development, ultimately enhancing the overall quality of patient care [10].

Revenue generation and reimbursement are integral components of financial management for hospitals. Administrators must navigate a complex landscape of healthcare reimbursement systems, including insurance billing, government programs, and private-payer agreements. Maximizing revenue while maintaining ethical billing practices is crucial for securing the financial stability to deliver high-quality care. These financial objectives are achieved through a variety of strategies. Hospitals often employ revenue cycle management strategies to streamline billing processes, reduce claims denials, and ensure timely reimbursement. Additionally, administrators may explore opportunities to diversify revenue streams, such as philanthropic donations or partnerships with other healthcare organizations [11].

Human Resources Management

Human resource management in healthcare is pivotal in shaping positive patient outcomes. It is imperative for hospital administrators to strategically manage their workforce to ensure that healthcare professionals are not only well-equipped but also motivated and engaged in delivering high-quality patient care [12]. In this context, the linchpin of effective human resource management lies in staffing and workforce planning. Hospital administrators are responsible for ensuring the hospital maintains an optimal mix of healthcare professionals, including physicians, nurses, allied health professionals, and support staff. Achieving this optimal mix and skill matching is essential for timely and effective patient care. Strategic workforce planning encompasses forecasting the hospital's staffing needs, which are influenced by patient volumes, acuity, and speciality requirements. By aligning staffing levels with patient demand, administrators can proactively address issues like understaffing or overstaffing, thereby optimizing resource utilization and ultimately improving patient outcomes [13]

Training and development are essential components of human resources management that contribute to improved patient outcomes. Continuous training and professional development programs are necessary to keep healthcare staff updated with the latest medical advancements, evidence-based practices, and patient-centered care approaches. Hospital administrators should invest in ongoing training initiatives that enhance clinical skills, communication abilities, and patient engagement techniques. By equipping healthcare professionals with the knowledge and tools they need to excel in their roles, hospitals can enhance the quality of patient care, ultimately leading to improved patient outcomes [14].

Employee satisfaction and retention are closely intertwined with patient satisfaction and patient outcomes. Hospital administrators must prioritize creating a supportive work environment that fosters a sense of belonging and purpose among healthcare staff. This includes offering competitive compensation packages, recognizing and celebrating the contributions of their staff, and providing opportunities for professional growth and career advancement. High levels of employee satisfaction can translate into lower turnover rates, which is essential for maintaining continuity of care and building a committed healthcare team dedicated to patient well-being. A satisfied and engaged workforce will likely go the extra mile to ensure exceptional patient care [15].

Quality Improvement and Patient Safety

Quality improvement and patient safety are integral components of hospital administration, and achieving these objectives necessitates implementing specific measures. These measures are crucial for enhancing patient outcomes and ensuring the delivery of safe, effective, and high-quality healthcare services. Hospital administrators are pivotal in driving these efforts forward [16].

Quality metrics and measurement are pivotal in evaluating and enhancing patient outcomes. Hospital administrators should establish comprehensive performance measurement systems to monitor various quality metrics. These metrics encompass clinical outcomes, adherence to best practices, patient satisfaction, and other related initiatives. They are indispensable tools for gauging the effectiveness of healthcare services and pinpointing areas that require enhancement. By consistently collecting and analyzing data about patient care, administrators can obtain valuable insights into the hospital's performance and, in turn, drive continuous quality improvement efforts. These efforts encompass a range of initiatives, which may involve reducing adverse events, refining clinical processes, and optimizing resource allocation to enhance patient outcomes [17].

Patient safety protocols and initiatives represent a fundamental aspect of hospital administration. Hospital administrators must establish and maintain robust safety protocols that address various aspects of patient care. This includes initiatives such as medication reconciliation, infection control measures, fall prevention programs, and implementing evidence-based clinical guidelines. By prioritizing patient safety, administrators can significantly reduce adverse events, medical errors, and patient harm. These efforts not only enhance patient outcomes but also contribute to building trust and confidence among patients and their families [18].

Continuous improvement methodologies, such as Lean Six Sigma or Plan-Do-Study-Act (PDSA) cycles, offer structured approaches to identify areas for improvement and implement evidence-based practices. Hospital administrators play a critical role in fostering a culture of continuous improvement throughout the organization. They can lead improvement teams, encourage staff engagement in quality improvement initiatives, and provide the necessary resources and support. Administrators should also ensure that data-driven decision-making is ingrained in the hospital's culture, enabling healthcare teams to identify and address issues promptly, make evidence-based adjustments to care processes, and ultimately achieve better patient outcomes [19].

Information Technology and Healthcare Systems

Electronic health records (EHR) and health information systems: Hospital administrators oversee the implementation and optimization of EHR and health information systems. EHRs facilitate digitizing patient records, enabling efficient clinical documentation, improving care coordination among healthcare providers, and providing clinicians instant access to critical patient information. By ensuring the effective utilization of EHRs, administrators support streamlined workflows, reduce medical errors, and enhance the overall quality and safety of patient care [20].

Telemedicine and healthcare technology trends: Telemedicine and emerging healthcare technologies are transforming the healthcare landscape. Hospital administrators must stay current with these trends and leverage technology to expand access to care, particularly in remote or underserved areas. Telemedicine enables virtual consultations, remote monitoring, and telehealth services, improving patient access to healthcare while enhancing convenience. Administrators can harness technology to improve patient engagement, facilitate communication between patients and providers, and promote remote health monitoring. Embracing healthcare technology trends enhances patient care and positions healthcare organizations for future growth and adaptability [21].

Data analytics and decision support systems: Data analytics and decision support systems empower hospital administrators to make informed, data-driven decisions. These tools enable the identification of trends and predictive analytics to anticipate patient needs and optimize resource allocation. Administrators should invest in robust data analytics infrastructure and foster a culture of data-driven decision-making within the organization. Data-driven insights can help improve clinical outcomes, optimize operational efficiency, and inform strategic planning. Hospital administrators should collaborate with data analysts and clinicians to derive actionable insights from healthcare data, ultimately leading to better patient outcomes and more efficient healthcare delivery [22].

Strategies for improving patient outcomes

Patient-centred care reimagines healthcare by prioritizing the patient in all decisions and actions. It tailors services to individual needs, values, and preferences, enhancing satisfaction and engagement. Enhancing satisfaction includes regular surveys for feedback, personalized care plans, minimizing wait times, effective patient education, respecting dignity, and patient advocacy programs. Effective communication is vital, with training in empathy and shared decision-making. Interdisciplinary care involves coordination, case conferences, information exchange, and standardized protocols. Team-based healthcare involves role clarity, digital platforms for collaboration, continuous education, patient-centred rounds, and quality improvement initiatives. Strategies for improving patient outcomes are in Table 1 [23-28].

**Table 1:** Strategies for improving patient outcomes

Strategy	Description
Patient-Centered Care	An approach that tailors care to meet individual patient needs and preferences, enhancing patient satisfaction and engagement.
Collaboration and Interdisciplinary Care	Promoting teamwork among healthcare professionals from different specialities for comprehensive and effective patient care.
Measurement and Evaluation	The use of key performance indicators and data analysis to monitor and assess patient outcomes, allowing for quality improvements.
Future Trends and Challenges	Staying informed about emerging technologies, evolving healthcare policies, and potential obstacles to adapt and thrive in the healthcare landscape.

Measurement and evaluation

Key Performance Indicators (KPIs) for Patient Outcomes

Mortality rates: Monitoring mortality rates is essential to assess the overall effectiveness of clinical care. Hospitals should establish robust mortality review committees that evaluate patient deaths to address this. These committees should analyze the root causes of mortality and implement quality improvement initiatives to prevent avoidable deaths. Interdisciplinary case conferences can facilitate collaborative discussions and learn from adverse events [29].

Readmission rates: High readmission rates can indicate issues with care quality and care transitions. Hospitals should implement care transition programs focusing on patient education, medication reconciliation, and follow-up care planning to address this. Regularly analyzing readmission data, identifying high-risk patient groups, and tailoring interventions to address their specific needs are crucial steps in reducing readmissions [30].

Complication rates: Measuring complication rates related to surgeries, medical interventions, and hospital-acquired infections is essential for patient safety. Hospitals should prioritize infection prevention and control measures, including hand hygiene, antimicrobial stewardship, and environmental cleaning protocols, to address this. Surgical teams should adhere to best practices and engage in regular surgical safety checklists. Comprehensive training and monitoring of healthcare-associated infections are vital for reducing complication rates [31].

Patient satisfaction scores: Gathering patient feedback through standardized surveys is essential for assessing the care experience. Hospitals should regularly administer patient satisfaction surveys and use the results to identify areas for improvement. Addressing low satisfaction scores may involve enhancing communication between patients and healthcare providers, improving pain management protocols, and enhancing staff responsiveness through training and workflow adjustments [24].

Length of stay: Assessing the average length of hospital stays for various conditions indicates care efficiency. Hospitals should implement care pathways and protocols that optimize patient flow and resource allocation to address this. Regular performance reviews and process improvement initiatives can help streamline care delivery and reduce unnecessary delays in patient discharge [32].

Patient safety indicators: Evaluating safety-related KPIs, such as falls with injury, medication errors, and pressure ulcers, is crucial for identifying improvement areas. Hospitals should establish patient safety committees investigating adverse events and near misses to address this. Root cause analyses should be conducted to identify systemic issues, and corrective actions should be implemented. Ongoing staff training on patient safety protocols is essential [33].

Quality of life metrics: Incorporating patient-reported outcomes measures (PROMs) helps assess the impact of care on patients' quality of life and well-being. To address this, hospitals should routinely administer PROMs to patients and use the data to tailor care plans and interventions. Healthcare teams should collaborate with patients to set realistic expectations and goals for improving quality of life [34].

Adherence to clinical guidelines: Measuring how clinical guidelines and best practices are followed in patient care is crucial for ensuring evidence-based treatment. To address this, hospitals should establish clinical guidelines committees responsible for developing, disseminating, and promoting adherence to evidence-based protocols. Regular audits and feedback mechanisms should be in place to monitor compliance with clinical guidelines and drive continuous improvement [35].

Data Collection and Analysis

Electronic health records (EHRs): EHR systems play a pivotal role in modern healthcare administration by providing a structured and standardized platform for collecting and storing patient data. To address this, hospitals should ensure the comprehensive adoption and proper utilization of EHR systems across all departments. Staff training and adherence to data entry protocols are essential to maintain data accuracy and consistency within EHRs. Regular updates and enhancements to EHR systems should also be prioritized to keep up with evolving healthcare requirements [36].

Data integration: Data integration is crucial for creating a comprehensive patient profile by aggregating information from various sources within the hospital. Hospitals should invest in data integration solutions that allow seamless data flow between EHRs, laboratory systems, billing records, and other sources. Data integration should be standardized to ensure data consistency and accuracy. Data governance policies should be established to maintain data quality and security [37].

Outcome tracking: Collecting data on patient outcomes, such as mortality, readmissions, and complications, is essential for evaluating hospital performance. Hospitals should establish a systematic outcome data collection and analysis process to address this. Real-time updates to outcome data should be prioritized to provide timely insights. Performance dashboards can visualize and track outcomes, enabling healthcare teams to make informed decisions and implement improvements [38].

Advanced analytics: Employing advanced analytics tools enables hospitals to analyze patient data in depth, identifying trends, correlations, and potential areas for improvement. Hospitals should invest in analytics software and train staff in data analysis techniques to address this. Collaboration between data analysts and clinical teams is essential to interpret and act on data-driven insights effectively. The results of advanced analytics should be communicated to stakeholders for informed decision-making [39].

Predictive analytics: Predictive modelling can anticipate patient needs and risks, enabling proactive interventions to prevent adverse outcomes. Hospitals should develop predictive analytics models tailored to their patient populations and clinical scenarios to address this. Regular updates and refinement of predictive models based on new data are necessary to maintain accuracy. Healthcare providers should be trained to interpret and act on predictive insights to improve patient care [40].

Real-time monitoring: Implementing real-time monitoring systems for critical patient metrics allows for immediate responses to deteriorating conditions. Hospitals should invest in monitoring technologies and establish protocols for continuous patient monitoring to address this. Staff should receive training on using monitoring systems effectively. Alarms and alerts should be configured to trigger timely interventions, and monitoring data should be integrated with EHRs for seamless data access and analysis [41].

Benchmarking and Comparing Hospital Performance

Peer benchmarking: Comparing key performance indicators with similar hospitals provides valuable insights into areas where improvement and best practices can be identified. Hospitals should establish relationships or partnerships with peer institutions, engage in data-sharing agreements, and participate in benchmarking studies to address this. Regular communication and collaboration with peer institutions are essential for mutual learning and improvement [42].

National and regional benchmarks: Benchmarking against national and regional benchmarks set by healthcare agencies and organizations provides a broader perspective on hospital performance. Hospitals should actively seek out these benchmarks and use them to measure their performance. To address this, hospitals should allocate data collection and analysis resources to ensure alignment with established benchmarks. They should also engage with industry associations and regulatory bodies to stay informed about relevant benchmarking standards [43].

Internal benchmarking: Evaluating the performance of different hospital departments or units within the organization effectively identifies disparities and shares best practices. To address this, hospitals should establish a culture of internal benchmarking that encourages departments to assess their performance against each other regularly. Cross-functional teams can facilitate knowledge sharing and collaboration between departments. Implementing performance improvement initiatives based on internal benchmarking findings is crucial for driving change [16].

Collaborative networks: Participating in collaborative healthcare networks and sharing data and experiences with other hospitals can lead to collective improvements in patient outcomes. Hospitals should actively seek out and join collaborative networks that align with their goals and patient populations to address this. They should contribute data, best practices, and insights to these networks and leverage the collective knowledge to implement evidence-based improvements [44].

Continuous monitoring: Regularly updating and reviewing benchmarking data is essential to track progress and adapt strategies accordingly. Hospitals should establish a robust data collection, analysis, and reporting system to address this. Automated data tracking and reporting tools can facilitate continuous monitoring. Regular performance reviews and strategy sessions should be conducted to ensure that benchmarking data is used effectively to drive ongoing improvement efforts [16].

Future trends and challenges

Emerging Technologies in Healthcare Administration

Artificial intelligence (AI) and machine learning: Integrating AI and machine learning in healthcare administration holds immense potential with several practical applications. Hospitals can consider investing in AI-driven tools to facilitate data analysis, predictive analytics, and administrative task automation. For instance, AI can predict patient admission rates, optimize staff schedules, and improve resource allocation. Additionally, AI can assist in claims processing, billing, and medical coding, reducing errors and increasing efficiency. To achieve successful integration, hospitals should create comprehensive AI implementation strategies. This includes identifying areas where AI can be most beneficial, setting performance metrics, and developing training programs for staff to utilize AI tools effectively. Ensuring data privacy and ethical considerations are upheld when implementing AI technologies is paramount, requiring robust data protection measures and adherence to ethical guidelines [45].

Blockchain: Blockchain technology offers a novel approach to healthcare data management, providing heightened security and transparency. Hospitals should explore blockchain applications in patient record-keeping, supply chain management, and billing processes. To address this technology, hospitals should collaborate with blockchain experts, implement robust blockchain solutions, and educate staff on blockchain principles. Ensuring compliance with data privacy regulations using blockchain is crucial to maintaining patient trust [46].

Telehealth and remote monitoring: The expansion of telehealth services and the adoption of remote monitoring devices represent a transformative trend in healthcare administration. Beyond the imperative statement that "they should", there are several compelling benefits and potential drawbacks to consider when evaluating the integration of these technologies. The benefits of telehealth and remote monitoring include improved patient access to care, especially for those in remote or underserved areas, reduced healthcare costs, and the ability to monitor and manage chronic conditions more effectively. Additionally, telehealth can enhance healthcare facility efficiency by reducing in-person visits and waiting times, and it offers the flexibility of virtual consultations, which can be particularly valuable during public health crises. However, it's crucial to acknowledge the potential challenges and drawbacks. Some healthcare facilities may hesitate to adopt telehealth due to concerns about data security and privacy, regulatory and licensing issues, and the need for substantial investments in technology and training. Moreover, not all medical conditions can be effectively addressed through virtual care, and there may be limitations to physical examinations and hands-on treatments. Infrastructure, financial resources, patient demographics, and regulatory frameworks influence the decision to embrace or resist telehealth technologies. It is essential to carefully assess these considerations in each healthcare facility's unique circumstances to make an informed decision on their implementation [47].

Robotic process automation (RPA): RPA can potentially optimize administrative tasks such as claims processing and appointment scheduling. Hospitals can benefit from the reduced error rates and increased efficiency that RPA offers. To address this, hospitals should identify areas where RPA can be applied effectively, implement RPA solutions, and provide training to staff working alongside automated processes. Regular monitoring and auditing of RPA systems are essential to ensure accuracy [48].

Internet of Things (IoT) in healthcare: IoT enables real-time monitoring of patients and medical equipment, contributing to patient safety and resource management. Hospitals should invest in IoT infrastructure, secure data transmission, and analytics capabilities. Critical considerations include addressing IoT security challenges and ensuring patient data remains confidential and protected [49].

Big data and analytics: Analyzing large volumes of healthcare data is invaluable for improving patient outcomes, resource allocation, and cost containment. Hospitals should invest in data analytics tools, hire skilled data analysts, and establish data governance policies. Additionally, hospitals should prioritize data privacy and security to maintain patient trust while using big data analytics [50].

Evolving Healthcare Policies and Regulations

Healthcare reform: Healthcare reform initiatives, including changes in reimbursement models, can significantly impact hospitals' financial sustainability and care delivery strategies. To address this challenge, hospitals should proactively adapt by diversifying their revenue streams, such as exploring value-based contracts and alternative payment models. This shift requires reevaluating care processes to prioritize quality outcomes, cost containment, and patient satisfaction. Hospitals should continuously monitor reform efforts, participate in advocacy efforts, and seek opportunities for collaboration with payers and other providers to align their strategies with evolving policies [51].

Privacy and data security: Compliance with data privacy regulations is paramount as healthcare becomes increasingly digital. The Health Insurance Portability and Accountability Act (HIPAA) sets stringent standards for safeguarding patient information in the United States. Hospitals must prioritize patient data protection by implementing robust cybersecurity measures, encryption protocols, and access controls. They should conduct regular security audits and risk assessments to identify vulnerabilities and ensure compliance. Additionally, staff training and awareness programs should emphasize the importance of data security, privacy best practices, and incident response protocols to prevent breaches [52].

Value-based care: The transition from fee-for-service to value-based care models necessitates a fundamental shift in hospital practices. Hospitals should focus on quality outcomes, population health management, and care coordination across the continuum. Hospitals should invest in care coordination teams, data analytics, and population health management tools to address this shift. Collaborative partnerships with primary care providers, specialists, and community organizations can help hospitals achieve better health outcomes and succeed in value-based contracts. Developing models prioritizing preventive care and early intervention is also essential [53].

International standards: Global hospitals must navigate a complex web of international healthcare regulations and standards. To address this challenge, hospitals should establish a comprehensive compliance strategy aligning with local and international standards. This includes understanding and adhering to regulations specific to each country of operation, ensuring cultural competence, and respecting regional ethical norms. Engaging with international healthcare organizations and staying informed about global healthcare trends and standards can aid hospitals in achieving compliance and maintaining a positive reputation on a global scale [54].

Health equity: Addressing health disparities and ensuring equitable care delivery is a growing imperative for healthcare policies. Hospitals should actively work toward reducing disparities by implementing programs that target underserved communities and addressing social determinants of health. This involves strategic resource allocation to support initiatives to reduce health disparities, such as community outreach, culturally competent care, and partnerships with local organizations. Hospitals should also collect and analyze data to identify disparities and measure progress toward achieving health equity [55].

Potential Obstacles and How to Address Them

Financial sustainability: The financial sustainability of hospitals is a fundamental challenge. Balancing the need for technological investments with limited resources can take time and effort. To address this challenge, hospitals should prioritize cost-effective solutions and strategic budgeting. This may involve conducting cost-benefit analyses to determine the most efficient use of funds, exploring partnerships with technology vendors for cost-sharing, and seeking grants or incentives for healthcare technology adoption [56].

Workforce adaptation: The rapid adoption of new technologies and care models may require healthcare staff to acquire new skills and adapt to changes in their roles. To address this, hospitals should develop comprehensive training programs that empower staff to leverage emerging technologies effectively. Continuous education and training initiatives can ensure that the workforce remains competent and confident in utilizing the latest tools and practices [57].

Interoperability: Achieving seamless data exchange between healthcare systems and technologies is crucial for effective patient care. Hospitals should advocate for standardized data formats and interoperable systems to overcome interoperability challenges. Collaborating with industry stakeholders and participating in interoperability initiatives is vital in this regard. A prime example of this is Epic Systems, which has demonstrated how adherence to established standards, such as Health Level Seven (HL7) and Fast Healthcare Interoperability Resource (FHIR), can facilitate data exchange and integration [58].

Cybersecurity: The digitization of healthcare introduces cybersecurity risks, including the threat of cyberattacks and data breaches that could compromise patient information. Hospitals should invest in robust cybersecurity measures, including firewalls, encryption, intrusion detection systems, and regular security assessments. Furthermore, staff training on cybersecurity best practices is vital to minimize human errors that may expose vulnerabilities [59].

Regulatory compliance: Healthcare regulations continually evolve, and hospitals must remain compliant to avoid legal and financial repercussions. Maintaining a dedicated compliance team is essential. Hospitals should also regularly monitor changes in healthcare regulations at the federal and state levels. Developing strong partnerships with legal and compliance experts can help hospitals stay updated and adapt swiftly to evolving policies [60].

Patient engagement: In telehealth and remote monitoring scenarios, engaging patients in their care can be challenging. Addressing barriers to technology adoption among specific patient populations, such as elderly or underserved communities, is essential. Hospitals should implement patient engagement strategies considering patients' diverse needs and preferences. This may involve providing education and training on technology use, offering user-friendly interfaces, and ensuring accessibility for all patients [61].

Healthcare disparities: Healthcare disparities persist among various demographic groups and communities, contributing to unequal patient outcomes. Hospitals should actively work to reduce these disparities by implementing programs that target underserved communities and address social determinants of health. Collaborating with community organizations, offering culturally sensitive care, and focusing on preventive care initiatives can contribute to more equitable healthcare delivery [62].

## Conclusions

In conclusion, this comprehensive review has underscored the undeniable significance of effective hospital administration in healthcare. It has illuminated the multifaceted nature of hospital administration, from the crucial role of leadership and the intricacies of financial management to the imperative of patient-centred care and interdisciplinary collaboration. Moreover, we have highlighted the essential role of data-driven measurement, analysis, and benchmarking in assessing hospital performance. Looking ahead, the future of healthcare administration is poised to be shaped by emerging technologies, evolving policies, and persistent challenges. However, amidst these changes, the resounding message is clear: effective hospital administration is pivotal in improving patient outcomes. Hospital administrators are not just stewards of institutions; they are architects of better healthcare, custodians of patient well-being, and quality champions. The call to action for healthcare leaders and policymakers is to prioritize the development of capable, forward-thinking administrators, invest in technology, promote value-based care, and address disparities to ensure that the promise of better patient outcomes remains at the heart of healthcare administration. Ultimately, the journey toward improved patient outcomes through effective hospital administration is a shared responsibility that holds the potential to transform healthcare for generations to come.

## References

[1] Kruk ME, Gage AD, Arsenault C (2018). High-quality health systems in the Sustainable Development Goals era: time for a revolution. Lancet Glob Health.

[2] Stoumpos AI, Kitsios F, Talias MA (2023). Digital transformation in healthcare: technology acceptance and its applications. Int J Environ Res Public Health.

[3] Manzoor F, Wei L, Hussain A, Asif M, Shah SI (2019). Patient satisfaction with health care services; an application of physician's behavior as a moderator. Int J Environ Res Public Health.

[4] Sfantou DF, Laliotis A, Patelarou AE, Sifaki-Pistolla D, Matalliotakis M, Patelarou E (2017). Importance of leadership style towards quality of care measures in healthcare settings: a systematic review. Healthcare (Basel).

[5] Babiker A, El Husseini M, Al Nemri A (2014). Health care professional development: working as a team to improve patient care. Sudan J Paediatr.

[6] Chanturidze T, Obermann K (2016). Governance in health - the need for exchange and evidence comment on "governance, government, and the search for new provider models". Int J Health Policy Manag.

[7] Varkey B (2021). Principles of clinical ethics and their application to practice. Med Princ Pract.

[8] Walters JK, Sharma A, Malica E, Harrison R (2022). Supporting efficiency improvement in public health systems: a rapid evidence synthesis. BMC Health Serv Res.

[9] (2023). Healthcare and Hospital Budgeting: A Complete Guide | Syntellis. Syntellis Perform. https://www.syntellis.com/guide-to-healthcare-and-hospital-budgeting.

[10] Akinleye DD, McNutt LA, Lazariu V, McLaughlin CC (2019). Correlation between hospital finances and quality and safety of patient care. PLoS One.

[11] Britton JR (2015). Healthcare reimbursement and quality improvement: integration using the electronic medical record comment on "fee-for-service payment--an evil practice that must be stamped out?". Int J Health Policy Manag.

[12] Hampel K, Hajduova Z (2023). Human resource management as an area of changes in a healthcare institution. Risk Manag Healthc Policy.

[13] Dubois CA, Singh D (2009). From staff-mix to skill-mix and beyond: towards a systemic approach to health workforce management. Hum Resour Health.

[14] CH M, SA M, EB A, AM A (2017). Empowering education: a new model for in-service training of nursing staff. J Adv Med Educ Prof.

[15] Karaca A, Durna Z (2019). Patient satisfaction with the quality of nursing care. Nurs Open.

[16] Hughes RG (2008). Tools and strategies for quality improvement and patient safety. Patient Safety.

[17] Quentin W, Partanen VM, Brownwood I (2019). Measuring Healthcare Quality. https://www.ncbi.nlm.nih.gov/books/NBK549260/.

[18] Ellenbecker CH, Samia L, Cushman MJ, Alster K (2008). Patient Safety and Quality in Home Health Care. Patient Safety.

[19] McDermott O, Antony J, Bhat S, Jayaraman R, Rosa A, Marolla G, Parida R (2022). Lean six sigma in healthcare: a systematic literature review on challenges, organisational readiness and critical success factors. Processes.

[20] Aguirre RR, Suarez O, Fuentes M, Sanchez-Gonzalez MA (2019). Electronic health record implementation: a review of resources and tools. Cureus.

[21] Jin MX, Kim SY, Miller LJ, Behari G, Correa R (2020). Telemedicine: current impact on the future. Cureus.

[22] Batko K, Ślęzak A (2022). The use of Big Data Analytics in healthcare. J Big Data.

[23] Fix GM, VanDeusen Lukas C, Bolton RE, Hill JN, Mueller N, LaVela SL, Bokhour BG (2018). Patient-centred care is a way of doing things: How healthcare employees conceptualize patient-centred care. Health Expect.

[24] Al-Abri R, Al-Balushi A (2014). Patient satisfaction survey as a tool towards quality improvement. Oman Med J.

[25] Birhanu Z, Abamecha F, Berhanu N, Dukessa T, Beharu M, Legesse S, Kebede Y (2021). Patients' healthcare, education, engagement, and empowerment rights' framework: patients', caretakers' and health care workers' perspectives from Oromia, Ethiopia. PLoS One.

[26] Hashim MJ (2017). Patient-Centered Communication: Basic Skills. Am Fam Physician.

[27] Bendowska A, Baum E (2023). The Significance of Cooperation in Interdisciplinary Health Care Teams as Perceived by Polish Medical Students. Int J Environ Res Public Health.

[28] Buljac-Samardzic M, Doekhie KD, van Wijngaarden JD (2020). Interventions to improve team effectiveness within health care: a systematic review of the past decade. Hum Resour Health.

[29] Stewart K, Choudry MI, Buckingham R (2016). Learning from hospital mortality. Clin Med (Lond).

[30] Kripalani S, Theobald CN, Anctil B, Vasilevskis EE (2014). Reducing hospital readmission rates: current strategies and future directions. Annu Rev Med.

[31] Mehta Y, Gupta A, Todi S (2014). Guidelines for prevention of hospital acquired infections. Indian J Crit Care Med.

[32] Baek H, Cho M, Kim S, Hwang H, Song M, Yoo S (2018). Analysis of length of hospital stay using electronic health records: a statistical and data mining approach. PLoS One.

[33] Ray B, Samaddar DP, Todi SK, Ramakrishnan N, John G, Ramasubban S (2009). Quality indicators for ICU: ISCCM guidelines for ICUs in India. Indian J Crit Care Med.

[34] Weldring T, Smith SM (2013). Patient-reported outcomes (PROs) and patient-reported outcome measures (PROMs). Health Serv Insights.

[35] Panteli D, Legido-Quigley H, Reichebner C, Ollenschläger G, Schäfer C, Busse R (2019). Clinical Practice Guidelines as A Quality Strategy. https://www.ncbi.nlm.nih.gov/books/NBK549283/.

[36] Ehrenstein V, Kharrazi H, Lehmann H, Taylor CO (2019). Obtaining Data from Electronic Health Records. Healthcare Research and Quality.

[37] Hoffmann K, Pelz A, Karg E (2023). Data integration between clinical research and patient care: a framework for context-depending data sharing and in silico predictions. PLOS Digit Health.

[38] Young M, Smith MA (2023). Standards and evaluation of healthcare quality, safety, and person-centered care. https://pubmed.ncbi.nlm.nih.gov/35015457/.

[39] Dash S, Shakyawar SK, Sharma M, Kaushik S (2019). Big data in healthcare: management, analysis and future prospects. J Big Data.

[40] Golas SB, Nikolova-Simons M, Palacholla R, Op den Buijs J, Garberg G, Orenstein A, Kvedar J (2021). Predictive analytics and tailored interventions improve clinical outcomes in older adults: a randomized controlled trial. NPJ Digit Med.

[41] Safavi KC, Driscoll W, Wiener-Kronish JP (2019). Remote surveillance technologies: realizing the aim of right patient, right data, right time. Anesth Analg.

[42] Wind A, van Harten WH (2017). Benchmarking specialty hospitals, a scoping review on theory and practice. BMC Health Serv Res.

[43] Ettorchi-Tardy A, Levif M, Michel P (2012). Benchmarking: a method for continuous quality improvement in health. Healthc Policy.

[44] Bosch B, Mansell H (2015). Interprofessional collaboration in health care: lessons to be learned from competitive sports. Can Pharm J (Ott).

[45] Bajwa J, Munir U, Nori A, Williams B (2021). Artificial intelligence in healthcare: transforming the practice of medicine. Future Healthc J.

[46] Saeed H, Malik H, Bashir U (2022). Blockchain technology in healthcare: a systematic review. PLoS One.

[47] Haleem A, Javaid M, Singh RP, Suman R (2021). Telemedicine for healthcare: capabilities, features, barriers, and applications. Sens Int.

[48] Davenport T, Kalakota R (2019). The potential for artificial intelligence in healthcare. Future Healthc J.

[49] Rejeb A, Rejeb K, Treiblmaier H, Appolloni A, Alghamdi S, Alhasawi Y, Iranmanesh M (2023). The internet of things (IoT) in healthcare: taking stock and moving forward. Internet Things.

[50] Raghupathi W, Raghupathi V (2014). Big data analytics in healthcare: promise and potential. Health Inf Sci Syst.

[51] McClellan M, Rajkumar R, Couch M (2021). Health care payers COVID-19 impact assessment: lessons learned and compelling needs. NAM Perspect.

[52] Mbonihankuye S, Nkunzimana A, Ndagijimana A (2019). Healthcare data security technology: HIPAA compliance. Wirel Commun Mob Comput.

[53] Teisberg E, Wallace S, O'Hara S (2020). Defining and implementing value-based health care: a strategic framework. Acad Med.

[54] Porter ME, Lee TH (2013). The strategy that will fix health care. Harv Bus Rev.

[55] National Academies of Sciences (2021). The Future of Nursing 2020-2030: Charting a Path to Achieve Health Equity. https://pubmed.ncbi.nlm.nih.gov/34524769/.

[56] Harris C, Green S, Elshaug AG (2017). Sustainability in Health care by Allocating Resources Effectively (SHARE) 10: operationalising disinvestment in a conceptual framework for resource allocation. BMC Health Serv Res.

[57] Booth RG, Strudwick G, McBride S, O’Connor S, Solano López AL (2021). How the nursing profession should adapt for a digital future. BMJ.

[58] Szarfman A, Levine JG, Tonning JM (2022). Recommendations for achieving interoperable and shareable medical data in the USA. Commun Med (Lond).

[59] He Y, Aliyu A, Evans M, Luo C (2021). Health care cybersecurity challenges and solutions under the climate of COVID-19: scoping review. J Med Internet Res.

[60] Committee on Quality of Health Care in America (2000). To Err Is Human: Building a Safer Health System. National Academies Press.

[61] Gajarawala SN, Pelkowski JN (2021). Telehealth benefits and barriers. J Nurse Pract.

[62] Jackson CS, Gracia JN (2014). Addressing health and health-care disparities: the role of a diverse workforce and the social determinants of health. Public Health Rep.

